# Global Trends of Benthic Bacterial Diversity and Community Composition Along Organic Enrichment Gradients of Salmon Farms

**DOI:** 10.3389/fmicb.2021.637811

**Published:** 2021-04-29

**Authors:** Larissa Frühe, Verena Dully, Dominik Forster, Nigel B. Keeley, Olivier Laroche, Xavier Pochon, Shawn Robinson, Thomas A. Wilding, Thorsten Stoeck

**Affiliations:** ^1^Ecology Group, Technische Universität Kaiserslautern, Kaiserslautern, Germany; ^2^Biosecurity, Coastal and Freshwater Group, Cawthron Institute, Nelson, New Zealand; ^3^Institute of Marine Research, Bergen, Norway; ^4^Institute of Marine Science, University of Auckland, Auckland, New Zealand; ^5^St. Andrews Biological Station, Department of Fisheries and Oceans, St. Andrews, NB, Canada; ^6^Scottish Association of Marine Sciences, Oban, United Kingdom

**Keywords:** aquaculture disturbance, bacterial community composition, bacterial diversity, community shifts, environmental DNA metabarcoding, environmental monitoring, organic enrichment, salmon farming

## Abstract

The analysis of benthic bacterial community structure has emerged as a powerful alternative to traditional microscopy-based taxonomic approaches to monitor aquaculture disturbance in coastal environments. However, local bacterial diversity and community composition vary with season, biogeographic region, hydrology, sediment texture, and aquafarm-specific parameters. Therefore, without an understanding of the inherent variation contained within community complexes, bacterial diversity surveys conducted at individual farms, countries, or specific seasons may not be able to infer global universal pictures of bacterial community diversity and composition at different degrees of aquaculture disturbance. We have analyzed environmental DNA (eDNA) metabarcodes (V3–V4 region of the hypervariable SSU rRNA gene) of 138 samples of different farms located in different major salmon-producing countries. For these samples, we identified universal bacterial core taxa that indicate high, moderate, and low aquaculture impact, regardless of sampling season, sampled country, seafloor substrate type, or local farming and environmental conditions. We also discuss bacterial taxon groups that are specific for individual local conditions. We then link the metabolic properties of the identified bacterial taxon groups to benthic processes, which provides a better understanding of universal benthic ecosystem function(ing) of coastal aquaculture sites. Our results may further guide the continuing development of a practical and generic bacterial eDNA-based environmental monitoring approach.

## Introduction

The aquaculture industry is becoming a major food production sector to satisfy the increasing global demand for fish and seafood, although the Western world is still developing its potential (FAO, 2020). Many marine finfish species, such as Atlantic salmon (*Salmo salar*), are typically grown in coastal open cage systems. Environmental impacts of finfish aquaculture on coastal ecosystems, especially benthic disturbance effects, are well-described (Holmer et al., 2008). These effects largely arise from the deposition of fish feces and uneaten food on the seafloor resulting in organic enrichment of sediments in the vicinity of fish farms (Carroll et al., 2003; Buschmann et al., 2006; Forrest et al., 2007; Wilson et al., 2009; Burridge et al., 2010). The vast majority of studies dedicated to the environmental impacts of coastal aquafarming analyzed either changes in benthic macrofaunal communities in the vicinity of salmon farms (Carroll et al., 2003; Macleod and Forbes, 2004; Porrello et al., 2005; Hall-Spencer et al., 2006) or the echoes of bacterial action through geochemical signals such as changes in oxygen and sulfide concentrations or in redox potential (Hargrave, 2010; Cranford et al., 2020). Such studies showed a succession of macrofaunal communities along organic enrichment gradients, generally following the traditional pattern described by Pearson and Rosenberg (1978): With increasing aquafarm-related benthic disturbance, benthic organisms that are less resistant to environmental change are replaced by fewer, more tolerant or opportunistic species. As the farm-related benthic disturbance decreases with increasing distance from the farming sites, the disturbance effect on macrofaunal communities co-decreases gradually (Brown et al., 1987; Keeley et al., 2012, 2013). Accordingly, regulatory environmental compliance monitoring programs take advantage of macroinvertebrates as bioindicators to assess the environmental quality of the seafloor in the vicinity of salmon farms (Black and Paterson, 1998; Borja et al., 2009; Sany et al., 2014).

Fewer studies investigated the benthic disturbance effects resulting from aquafarming on benthic bacterial communities. Bacteria are key players for nutrient (re)cycling and the (re)mineralization of organic matter. In particular, bacterial metabolism is responsible for the breakdown of organic deposits and harmful compounds discharged from salmon farms, consuming most, if not all, the available oxygen in the sediment pore water. The resulting metabolites, toxic gases, oxygen depletion, and acidification alter the benthic ecosystem, leading to pronounced changes in bacterial community structure (McCaig et al., 1999; Asami et al., 2005; Kawahara et al., 2009; Fodelianakis et al., 2014) and also drive the distribution patterns typically observed for macroinvertebrates (Christensen et al., 2000; Holmer et al., 2003; Bissett et al., 2006, 2007, 2009; Garren et al., 2008). However, reported results of bacterial diversity surveys in salmon farm sediments are often incongruent. For example, some studies reported that bacterial richness was higher at farm-impacted sites compared with reference sites (Powell et al., 2003; Bissett et al., 2007; Marcial Gomes et al., 2008), while others reported no difference between these two categories (Torsvik et al., 1996; Zhang et al., 2008; Kawahara et al., 2009). A study by Hornick and Buschmann (2017) even found slightly lower bacterial operational taxonomic unit (OTU) richness at some aquaculture site sediments compared with reference samples. Regarding bacterial community composition, reports are similarly heterogeneous. For example, some studies reported sulfate-reducing bacteria (SRBs) as a dominant and characteristic feature of benthic bacterial communities at aquaculture sites (Bissett et al., 2006; Dowle et al., 2015), while others found only negligible contributions of SRBs to benthic bacterial communities in the vicinity of salmon cages (Hornick and Buschmann, 2017). The main reason for such contrasting reports is likely the enormous complexity of factors shaping bacterial diversity and community composition at aquaculture sites. Marine sediments, in general, function as a reservoir of absorbed nutrients, heavy metals, pesticides, and other toxic compounds and are affected by geological, hydrological, physicochemical, and biotic interactions (Burdige, 2020). The structures and metabolism of benthic bacterial communities are sensitive to these (combinations of) parameters (Allison and Martiny, 2008). Aquaculture adds to these effects in complex ways through the discharge of a variety of compounds into the receiving benthic system (Holmer et al., 2008). In addition, the diversity and composition of local bacterial communities can also depend on the geographic region (Martiny et al., 2006; Fodelianakis et al., 2014), natural seasonal events (Navarro et al., 2008), and sediment texture, which again are influenced by local hydrological and geological conditions (Black et al., 1996; Sweetman et al., 2014). Due to the intrinsic variability in microbial communities, a detailed spatio-temporal investigation over larger sample sets is therefore required to obtain a proper understanding of general trends in bacterial diversity and community composition shifts along an impact gradient from aquaculture sites, which remain obscured in smaller datasets from individual farms, seasons, or geographic regions (Hornick and Buschmann, 2017). Elucidating such general patterns of bacterial diversity and community structures is key for a better understanding of benthic ecosystem effects resulting from aquafarming (Bissett et al., 2006; Castine et al., 2009). Eventually, such a knowledge will foster the development of a DNA-based bioindicator tool to monitor the environmental impacts, subsequent recovery, and relative performance of marine coastal aquaculture (Stoeck et al., 2018a, b; Verhoeven et al., 2018; Keeley et al., 2019; Armstrong and Verhoeven, 2020; Frühe et al., 2020).

Here, we analyzed bacterial environmental DNA (eDNA) metabarcoding datasets from 138 samples collected at different seasons, reflecting diverse farming conditions, different bottom substrates, water flow regimes, and water depths and originating from different biogeographic regions (Scotland, Norway, Canada, and New Zealand). Our study reveals common trends in bacterial diversity and community composition shifts from salmon cage sites to less impacted transition zones and predicted non-impacted reference sites, which are independent of the many environmental parameters that may change from farm to farm. We then link the metabolic properties of the observed bacterial communities to typical (universal) aquaculture disturbance.

## Materials and Methods

### Sampling Sites and Sample Classification

We analyzed 138 sediment samples collected from 10 Atlantic salmon (*Salmo salar*) farms in Norway (farms Aukrasanden, Bjørnsvik, and Nedre Kvarv), New Zealand (Big Glory Bay, Otanerau, and Te Pangu), Scotland (FH, MAC, and TG), and Canada (Crow Island). Sampling was conducted by contractors or by aquafarming companies for regulatory compliance monitoring to assess environmental quality at aquaculture sites. Benthic sampling for Scottish, Norwegian, and New Zealand samples was conducted as described previously (Cordier et al., 2018; Keeley et al., 2018; Dully et al., 2020) using van Veen grabs. Classification of samples into their ecological status occurred in the context of compliance monitoring through microscopic identification of macroinvertebrate bioindicators and calculation of the biotic index AZTI Marine Biotic Index (AMBI), based on which the ecological status was assigned to samples as described in Muxika et al. (2005). For our study, we then sorted samples into three aquaculture disturbance categories as shown in Table 1: (a) a high impact category, which united all samples (*n* = 31) with poor-to-bad ecological status (AMBI 4.3 < AMBI ≤ 7.0); b) a moderate impact category (*n* = 54), including all samples with moderate ecological status (AMBI 3.3 < AMBI ≤ 4.3); and (c) a low impact category, which included all samples (*n* = 53) with good-to-high ecological status (AMBI 0.0 < AMBI ≤ 3.3). For Canadian samples, macrofauna-based monitoring results were not available. Therefore, we tentatively assigned ecological status to these samples based on distance from the cages and previous compliance monitoring results from the Canadian Crow Island salmon farm. Further details of the sampling sites are provided as a Supplementary File 1.

**TABLE 1:** Overview of 138 samples from each farm under study and their assignments to aquaculture-related impact categories (high, moderate, and low).

Country of origin	Farm	High impact	Moderate impact	Low impact
Canada	Crow Island	4	12	6
Norway	Aukrasanden	3	0	6
Norway	Bjørnsvik	3	5	3
Norway	Nedre Kvarv	5	3	6
New Zealand	Big Glory Bay	3	6	6
New Zealand	Otanerau	3	3	9
New Zealand	Te Pangu	3	6	6
Scotland	FH	3	6	4
Scotland	MAC	2	6	4
Scotland	TG	2	6	4

*For definition of categories, see “Materials and Methods” section. Impact category was obtained from regulatory compliance monitoring data of benthic macroinvertebrate indicators.*

### Sampling of Environmental DNA

Sediment samples used for bacterial eDNA metabarcoding (Table 1) were collected from the van Veen grabs of the original regulatory compliance monitoring that was conducted by professional companies. The number of replicates analyzed varied between farms and countries due to different national compliance monitoring regulations or due to difficulties during sampling (e.g., due to seafloor conditions, sediment structure, or weather conditions; sometimes, only two replicated van Veen grabs could be collected successfully).

For Norwegian samples, we obtained extracted DNA from the Pawlowski Lab (University of Geneva, Switzerland). New Zealand, Scottish, and Canadian samples were original sediments that were further processed as described below. Samples from the Canadian farm were collected along transects radiating outward from the farm with six replicate syringe cores at each station by divers in the soft-bottom sediments as part of a larger aquaculture monitoring program. The cores were capped after collection, placed on ice, transported back to the lab where subsamples were taken of the surface layer (top 1 cm), and placed in sterile containers. All samples were preserved in LifeGuard isolation (Qiagen, Hildesheim, Germany) solution prior to further processing in the laboratory.

### DNA Extraction, PCR Amplification, and High-Throughput Sequencing

Total eDNA for all samples was obtained from homogenized sediment using Qiagen’s DNeasy PowerSoil kit according to the manufacturer’s instructions. PCR amplification of the hypervariable V3–V4 region of the bacterial SSU rRNA gene for all samples employed the protocol described by Herlemann et al. (2011) using Bakt_341F (CCTACGGGNGGCWGCAG) and Bakt_805R (GACTACHVGGGTATCTAATCC) as PCR primers. Initial activation of the Phusion High-Fidelity DNA polymerase (New England Biolabs) took place at 98°C for 30 s, followed by 27 identical cycles with three steps (98°C for 10 s, 62°C for 30 s, and 72°C for 30 s), and a final elongation at 72°C for 5 min. Sequencing libraries were constructed from PCR products using the NEB Next^®^ Ultra^TM^ DNA Library Prep Kit for Illumina (NEB, United States). Quality of libraries was verified using an Agilent Bioanalyzer 2100 by checking sizes, quantity, and purity of PCR enrichments. Libraries were sequenced on an Illumina MiSeq platform, generating 2 × 250-bp paired-end reads (SeqIT GmbH & Co. KG, Kaiserslautern, Germany). Sequence data are available at NCBI’s Sequence Read Archive under the following bioproject numbers: PRJNA562304 (Aukrasanden, Nedre Kvarv, and Bjørnsvik), PRJNA666153 (New Zealand), PRJNA666305 (TG, FH, and MAC), and PRJNA666128 (Crow Island).

### Sequence Data Processing and Taxonomic Assignment

Illumina sequencing adapters and primers were removed with cutadapt v.2.11.0 (Martin, 2011). With the use of DADA2 (Callahan et al., 2016), sequences were filtered, trimmed, and clustered (parameters: truncLen = 230, maxEE = 1, minOverlap = 20, maxMismatch = 2) into amplicon sequence variants (ASVs). The truncation length criterion was determined by choosing the sequence position at which Phred assigned a quality score of ≥30 (Q3) for at least 51% of all reads in a dataset (=base call accuracy 99.9%) (Ewing and Green, 1998). For maxEE we chose the most stringent value. We chose these settings to maximize the quality of the final sequence reads used for downstream analyses. Bacterial V3–V4 sequences were merged using 20-bp overlap with an allowed mismatch of 2. Chimeras were removed with *–uchime_denovo* function for vsearch (Rognes et al., 2016). Taxonomy was assigned to ASVs by comparing the sequences with the sequences stored in the Greengenes reference database (McDonald et al., 2012) using a last common ancestor approach (*–sintax* function of vsearch). Sequences that could not be assigned to the domain Bacteria were discarded. Also, ASVs with a read abundance of one were eliminated from the ASV-to-sample matrix to reduce noise.

### Community Statistics

All community statistical analyses were conducted using R v.3.6.3. The ASV-to-sample matrix was normalized (relative abundance) using the *decostand* function of R-package vegan v.2.5-5 (Oksanen et al., 2020) to account for uneven sampling sizes (read numbers and sample saturation, Supplementary File 2). Rarefied ASV richness, Shannon index (H’) (Shannon, 1949; Simpson, 1949), and Pielou’s evenness (J) (Pielou, 1966) were calculated as measures of alpha diversity using the *diversity* function of the R-package vegan v.2.5-5 (Oksanen et al., 2020). First, we tested for non-normal distribution of data with the Shapiro–Wilk test using *shapiro.test* function of R-package stats v.4.0.3 (Shapiro and Wilk, 1965). Significance of differences in alpha diversity measures among the cage sites, the transition zones, and the reference sites were tested using Kruskal–Wallis test (Kruskal and Wallis, 1952) using the *kruskal.test* function of the R-package stats v.4.0.3 followed by *post-hoc* pairwise Wilcoxon rank sum test using *pairwise.wilcox.test* function from the same package to calculate pairwise comparisons between enrichment categories with corrections for multiple testing. Prior to this, a permutational multivariate ANOVA (PERMANOVA) using the *adonis* function within the R-Package vegan was conducted to verify that farm site has a stronger effect than distance on microbial community structures.

### Distribution of Bacterial Taxa Across Different Organic Enrichment Categories

The identification of universal distribution patterns of individual bacterial taxa was conducted in two steps. In the first step, we identified the bacterial taxonomic rank, which was the most discriminant for high, moderate, and low aquafarm-related impact, using random forest (RF) classification. These classifications were conducted for the following taxonomic ranks: phylum, class, order, family, genus, and species. For RF analyses, we used the R-package randomForest v.4.6-14 (Breiman, 2001; Liaw and Wiener, 2002). Prior to RF analysis, the ASV-to-sample matrix was filtered for ASVs with a read abundance of at least 50, resulting in 8,740 bacterial ASVs. The motivation of this step was to reduce low-abundant reads, which are meaningless for classifications. The read counts were then normalized using fourth root transformation. The RF classification algorithm was tuned using different *m* (mtry) values (square root number of variables for classification ± 3), and the number of decision trees (ntree) was increased until the out-of-bag error (OOB error) had reached its minimum. Variable importance for the most powerful predictive taxon groups within the most informative taxon rank was calculated with mean decrease accuracy (MDA). The decrease in accuracy is measured by permuting the values of the predictor in the OOB samples and calculating the corresponding decrease. MDA is an important measure, which informs about the variables (here: taxon groups within the most powerful taxon rank) associated with the response (here: degree of organic enrichment) after modeling the data (Breiman, 2001). Agreement between predicted (based on bacterial taxon ranks) and observed (based on macroinvertebrate surveys) impact categories was tested using the *confusionMatrix* function of the caret v.6.0-86 R package. Agreement between the two classifications was considered as “poor agreement” (i.e., kappa value ranging from 0.01 to 0.2) to “almost perfect agreement” (i.e., kappa value ranging from 0.8 to 1) (Landis and Koch, 1977a, b).

In the second step, we then applied a permutation test (Pitman, 1938) in R to reveal significant differences in the distribution of the most powerful predictive taxon rank (obtained from RF classification) across the three classification categories (high, moderate, and low impact). For the permutation test, we compared the difference between means of the observed dataset against differences between means from 1,000 permutations of the dataset, in which the labels where randomly shuffled. To only consider taxa that show a universal rather than random pattern, we only included taxa in significance testing, which occurred in more than 51% of all samples within at least one of these three categories.

## Results

### Sequence Data Overview

Overall, 5,326,942 high-quality reads were obtained after sequencing, quality filtering, and taxonomic assignment, which were used for downstream analyses. These sequences clustered into 125,749 ASVs, 2,536 (1.8%) of which could not be assigned to a phylum and were termed “unclassified bacteria.” The remaining ASVs could be assigned to 28 different phyla. Only 1,512 ASVs (1.2%) could be taxonomically assigned down to species level. Supplementary File 3 details the sequence data for each individual farm under study.

### Alpha Diversity

**FIGURE 1:**
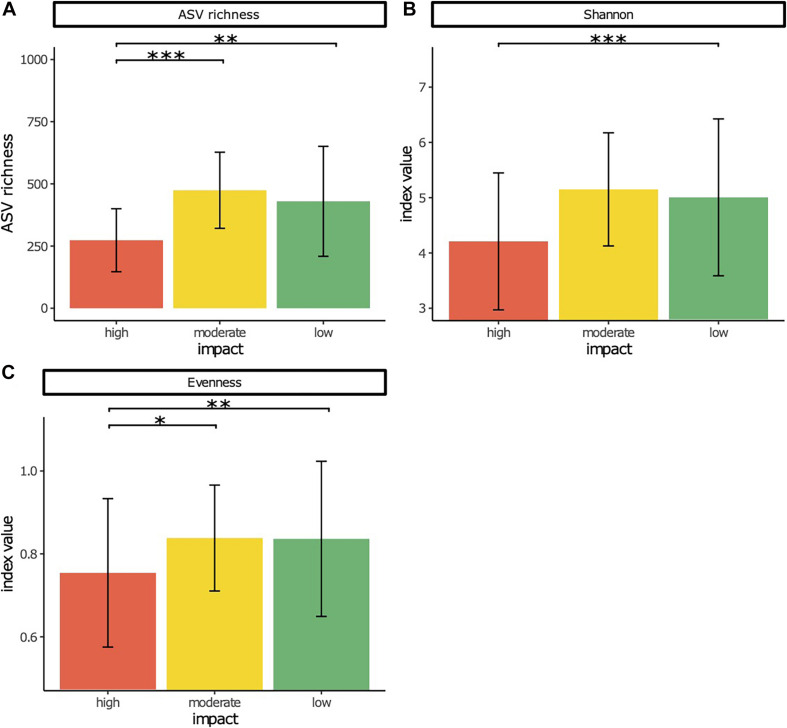
Rarefied amplicon sequence variant (ASV) richness **(A)**, Shannon diversity **(B)**, and Pielou’s evenness **(C)** as measures of alpha diversity of bacterial communities in sediment samples from high, low, and moderate salmon aquaculture-related impact. Significant differences are indicated by * (*p < 0.05, **p < 0.01, ***p < 0.001).

Bacterial ASV richness (Figure 1A) was significantly lower at high impact sites compared with moderate and low impact sites (*p* < 0.001 and *p* < 0.01, respectively), whereas Shannon diversity was significantly lower at high impact sites than at reference sites (*p* < 0.001) (Figure 1B). No significant differences were obtained between moderate and low impact sites for both measures. Pielou’s evenness (Figure 1C) was significantly lower at high impact samples compared with moderate (*p* < 0.05) and low impact (*p* < 0.01) samples.

### Predictive Performance of Taxonomic Ranks for the Degree of Organic Enrichment

Using RF classification, we calculated the prediction accuracy, with which individual taxon ranks succeeded to indicate the origin of a sample (cage sites, transition zones, and reference sites) and, thus, the degree of aquaculture-related benthic disturbance (Table 2). The combination of the highest prediction accuracy (in%) paired with the highest kappa value identified the family rank (kappa 0.78, accuracy 85.51%) as the most powerful taxonomic entity, followed by the order rank (kappa 0.72, accuracy 81.88%) and class rank (kappa 0.71, accuracy 81.16%). Genus rank (kappa 0.63, accuracy 76.09%) and phylum (kappa 0.58, accuracy 72.46%) were less powerful. The by far least-informative taxon rank to predict the sample origin was the species rank (kappa 0.03, accuracy 39.89%). Because the family rank has the highest prediction accuracy, all following results are based on the family rank as the most powerful taxon rank to predict sample origin (aquaculture-related impact category).

**TABLE 2:** Accuracy of prediction of high, moderate, and low aquaculture-related impact using bacterial taxon ranks as features and impact category (obtained from macrofauna surveys) as reference data.

Taxon rank	N features	Accuracy (%)	Kappa
Phylum	22	72.46	0.58
Class	38	81.16	0.71
Order	63	81.88	0.72
Family	88	85.51	0.78
Genus	68	76.09	0.63
Species	7	39.89	0.03

*Agreement between predicted (based on bacterial taxon ranks) and observed (based on macroinvertebrate surveys) impact categories is indicated by the kappa value, which ranges from “poor” (i.e., kappa value ranging from 0.01 to 0.2) to “almost perfect” (i.e., kappa value ranging from 0.8 to 1) (Landis and Koch, 1977a, b).*

### Random Forest Predictors for the Degree of Organic Enrichment

**FIGURE 2:**
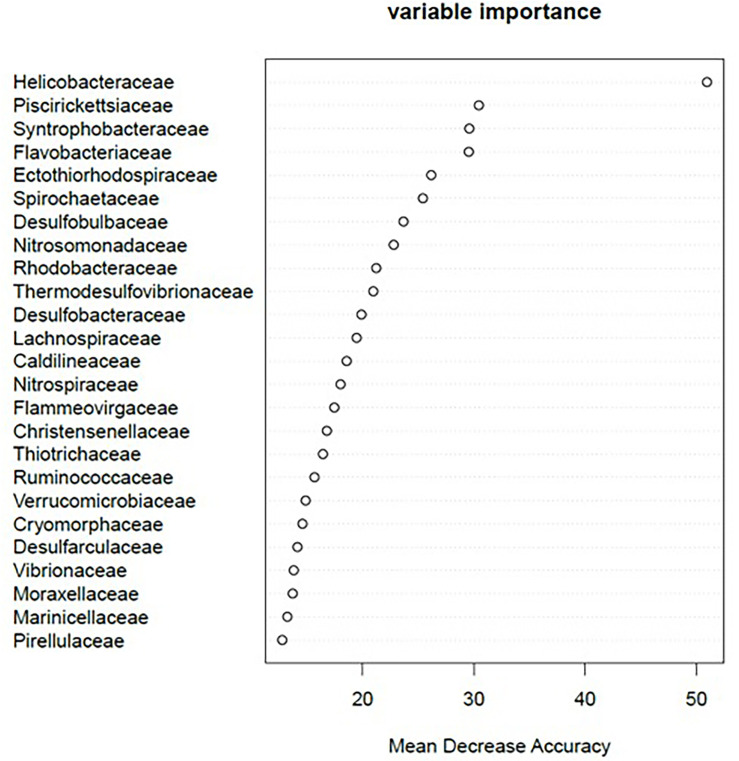
Variable importance of the 25 most powerful bacterial families to predict the degree of aquaculture-related impact using random forest. The complete list of variable importance and corresponding Gini coefficient for each of the 88 bacterial families identified in this study is available as Supplementary File 4.

The 25 strongest predictors (bacterial families) to infer the degree of aquaculture impact from the RF model are presented in Figure 2. The most discriminant bacterial families associated with the response to aquaculture impact were Helicobacteraceae, Piscirickettsiaceae, Syntrophobacteraceae, Flavobacteriaceae, Ectothiorhodospiraceae, Spirochaetaceae, Desulfobulbaceae, and Nitrosomonadaceae. Mean decrease accuracy and Gini coefficient of all 88 families are given as Supplementary File 4.

### Distribution of Bacterial Families Across the Organic Enrichment Categories

Bacterial families that were exclusive to the high (*n* = 5), moderate (*n* = 6), and low impact sites (*n* = 23) are presented in Supplementary File 5. The vast majority of these exclusive families occurred only in very few of the samples within their respective impact category.

**FIGURE 3:**
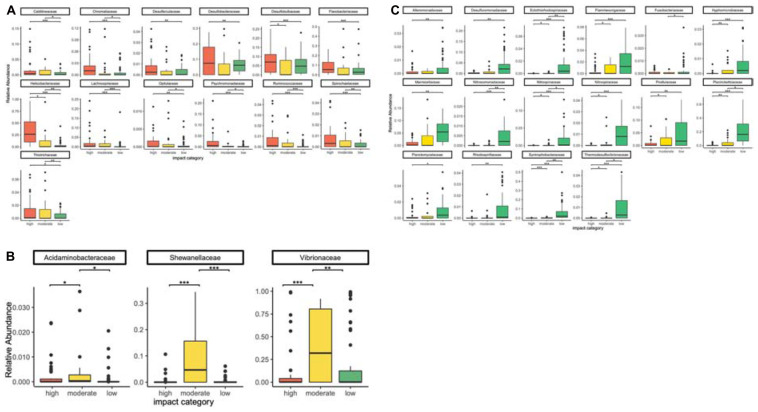
Differential occurrence of bacterial families with the highest relative abundances in high **(A)**, moderate **(B)**, and low **(C)** aquaculture-related impact samples. Significant differences (Pitman’s permutation test) are indicated by * (*p < 0.05, **p < 0.01, ***p < 0.001).

We found 89 bacterial families that were distributed ubiquitously across all sampling categories (Supplementary File 6). Of these, 38 occurred in at least 51% of the samples within an impact category. Permutation significance testing identified 32 of these 38 families with a significant differential occurrence in high (*n* = 13; Figure 3A), moderate (*n* = 3; Figure 3B), or low impact samples (*n* = 16; Figure 3C). With very few exceptions, these 32 families occurred in all sampled biogeographic regions. The only exceptions were six families, which were not found in the Canadian bottom substrate (Supplementary File 7). Of these 32 families, 20 were also among the 25 strongest RF predictors for sample origin (Figure 2).

**FIGURE 4:**
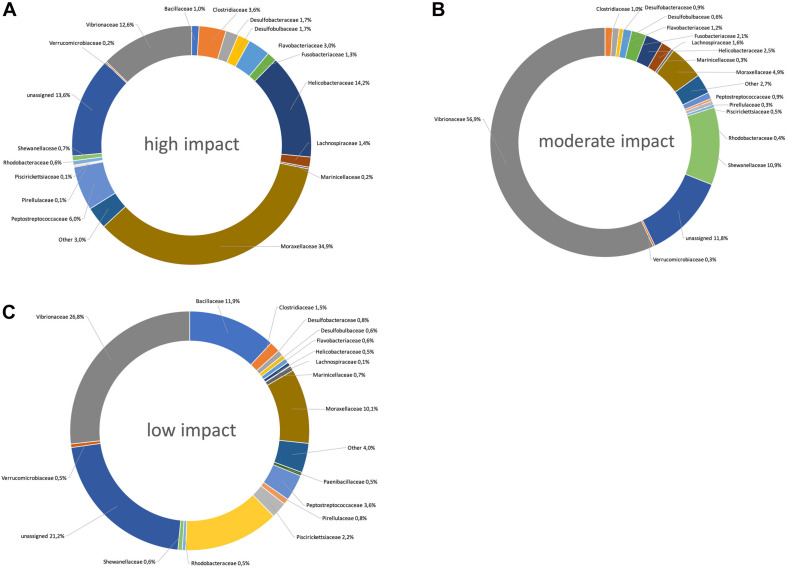
Pie charts showing the taxonomic distribution (relative abundances) of bacterial taxon ranks at high **(A)**, moderate **(B)**, and low **(C)** aquaculture-related impact sites.

Bacterial families with a significantly higher relative abundance in high impact samples were Caldilineaceae, Desulfarculaceae, Desulfobacteraceae, Desulfobulbaceae, Flavobacteriaceae, Helicobacteraceae, Lachnospiraceae, Psychromonadaceae, Ruminococcaceae, Spirochaetaceae (all of the former also belonged to the 25 most powerful RF predictors for sample origin, Figure 2), Chromatiaceae, Opitutaceae, and Thiotrichaceae (Figures 3A, 4A).

Only three bacterial families were significantly more abundant at moderately impacted sites, which were Acidaminobacteraceae, Vibrionaceae, and Shewanellaceae (Figures 3B, 4B). Vibrionaceae ranked among the 25 most powerful RF predictors (Figure 2).

Families with a significantly higher relative abundance at sites with low aquaculture impact (Figures 3C, 4C) were Ectothiorhodospiraceae, Flammeovirgaceae, Marinicellaceae, Nitrosomonadaceae, Nitrospiraceae, Pirellulaceae, Piscirickettsiaceae, Syntrophobacteraceae, Thermodesulfovibrionaceae (all of which belonging to the 25 most powerful RF predictors), Alteromonadaceae, Desulfuromonadaceae, Fusobacteriaceae, Hyphomicrobiaceae, Nitrospinaceae, Planctomycetaceae, and Rhodospirillaceae.

## Discussion

### Bacterial Diversity

The analyses of DNA metabarcodes from 138 samples obtained from 10 distinct salmon farms in four different countries (Norway, Scotland, New Zealand, and Canada) identified common trends in bacterial diversity patterns and bacterial taxa occurring in sediments subjected to different degrees of aquaculture disturbance (high, moderate, and low aquaculture impact).

Aquatic microbes and their metabolism are highly sensitive to environmental stressors, and previous studies demonstrated that functionally intact ecosystems with a high heterogeneity of the resource pool have diverse and relatively evenly distributed microbial communities and functions (Horton et al., 2019; Liao et al., 2019; Muscarella et al., 2019). Therefore, changes in diversity, richness, and evenness of microbial communities along environmental gradients can be considered as indicators of disturbance effects and, more specifically, as indicators of organic matter composition and environmental conditions related to oxygen (Horton et al., 2019). Previous reports about alpha diversity shifts of microbial communities from highly impacted aquaculture sites to less impacted reference sites are contrasting (Torsvik et al., 1996; Karakassis and Hatziyanni, 2000; Powell et al., 2003; Bissett et al., 2007; Marcial Gomes et al., 2008; Zhang et al., 2008; Kawahara et al., 2009; Hornick and Buschmann, 2017). This is explained by the complex interactions of a multitude of environmental parameters that act on bacterial diversity (Hornick and Buschmann, 2017). It also accounts for the relatively high standard deviation in alpha diversity measures in our analyses of 138 samples from 10 distinct salmon farms (Figure 1). Nevertheless, the general trend that emerged from this larger-scale study is an increase in diversity measures (ASV richness, Shannon diversity, and Pielou’s evenness) from high impact to low impact sites. Based on this finding, we can conclude that with increasing aquaculture impact, the benthic ecosystem is increasingly disturbed.

Stressors from aquaculture sites that may negatively influence bacterial diversity include the following: (i) sediment texture and proportion of fine particles in the sediment, which is usually notably higher in aquaculture sediments sites compared with far-field reference sites. The interplay of sediment texture, organic matter (availability), oxygen, and microbial community diversity has been widely reported; and for a detailed discussion, we refer to, e.g., Böer et al. (2009), Wang et al. (2013), and Zheng et al. (2014). In brief, sediment texture is a significant determinant for sediment water content, nutrient availability, and retention and, thus, is an essential property, which affects the ability of microbes to access organic substances. Additionally, adhesion capabilities of bacteria to attach to different sediment textures play an important role (Dang and Lovell, 2016) to structure the diversity of benthic bacterial communities. In general, bacterial diversity is lower in sediments with increasing proportions of fine particles (<63 μm) (Stoeck and Albers, 1999, 2000; Stoeck and Kröncke, 2001). (ii) In addition, metal contamination and other chemical substances released from fish farms such as feed supplements may add to benthic disturbance effects (Yeats et al., 2005; Holmer et al., 2008; Burridge et al., 2010) and alter diversity of bacterial communities in receiving fish farm sediments (Hornick and Buschmann, 2017). (iii) Metabolites and toxic gases such as hydrogen sulfide originating from the bacterial degradation of organic matter affect ecosystem properties (Holmer and Kristensen, 1996; Hargrave, 2010) and require highly adapted bacterial communities (Holmer et al., 2005). This may limit diversification in sediments close to the farming site. In contrast, sediments with lower organic enrichment provide a higher habitat heterogeneity with many microniches, fostering a diversification of bacterial communities and their metabolic functions. For example, the increasing size and bioturbation activity of macroinvertebrates from salmon cage sites toward less impacted reference sites (Nickell et al., 2003; Holmer et al., 2005) foster microbial activity, diversity, and interactions, predominantly through the formation of microenvironments (Bertics and Ziebis, 2009). (iv) The use of medication to treat fish in individual farms, such as emamectin benzoate used to protect salmon from the parasitic sea lice (Wilding and Black, 2015; Bloodworth et al., 2019), which accumulate in sediments around fish farms, may also cause lower bacterial diversity than in non-contaminated sediments.

### Bacterial Community Composition

Bacterial community composition might be more sensitive than the richness and diversity to the studied environmental conditions (Zheng et al., 2014). Therefore, a detailed analysis of the differential relative abundance patterns of individual bacterial taxon groups provides further information regarding the influence of salmon aquaculture on bacterial communities and the ecology of the salmon farm-exposed seafloor. Such compositional data analysis is a challenge in community statistics, when datasets are highly dimensional, as is typically the case when analyzing bacterial communities in complex environments such as marine sediments. The number of taxa is usually notably higher than the number of samples and also sparse, because numerous taxa occur in a small number of samples. For example, in our ASV-to-sample matrix, the sparsity is 98%, and even after collating the matrix across the family rank, three quarters of the data in the matrix are zeros (sparsity 74%). Despite sophisticated approaches to statistically transform such data, the analysis of compositional data remains a partially intractable problem, and the scientific community has not agreed to give consent (reviewed in Tsilimigras and Fodor, 2016). Therefore, we here have used two different commonly applied approaches for microbiome datasets, namely, fourth root and centered log-ratio (clr) transformation. Both methods are highly congruent in their results (for a direct comparison, see Supplementary File 8).

For the inference of ecological quality in the framework of eDNA-based environmental monitoring of aquaculture disturbance, ASVs are the preferred choice (Pawlowski et al., 2016; Verhoeven et al., 2016, 2018; Cordier et al., 2017, 2018; Keeley et al., 2018; Stoeck et al., 2018a,b; Forster et al., 2019; Armstrong and Verhoeven, 2020; Dully et al., 2020; Frühe et al., 2020). This is because of the higher resolution of individual ASVs compared with taxonomic ranks and because biotic indices can be calculated from ASVs without having any ecological information about these ASVs. This, again, is made possible by the application of specific analytical tools such as supervised machine learning (Cordier et al., 2018; Cordier, 2020) or quantile regression splines analyses (Keeley et al., 2018).

However, without a proper taxonomic assignment, the ecological information included in ASVs is very limited (Aylagas et al., 2016; Cordier et al., 2017). Because the aim of our study was to link the ecological properties of bacterial taxon groups to the degree of aquaculture impact on the benthic environment, we have refrained from analyzing individual bacterial ASVs in detail but instead considered ecologically informative taxon ranks. Among all taxon ranks analyzed as discriminators for the degree of aquaculture impact on the benthic environment, the species rank had by far the lowest predictive performance. Obviously, the major reason is the incompleteness of bacterial reference databases that are used for the taxonomic classification, especially on the species rank (Beng and Corlett, 2020). This emphasizes the strength of taxonomy-free approaches in eDNA-based environmental monitoring, which also considers important indicators, which cannot be taxonomically assigned.

### Bacterial Families Typical of High Impact Sites

Helicobacteraceae, which showed the most pronounced shifts between sites and a prominent occurrence at high impact sites, were identified previously as bioindicators for anthropogenically impacted coastal marine sediments, which exceeded the Sediment Quality Guidelines of the European Water Framework Directive for organic compounds (Menchaca et al., 2014) and which had high concentrations of organic matter content (Aylagas et al., 2017). These authors classified Helicobacteraceae as bioindicators of moderate-to-poor ecological status of marine sediments. Several members of this family are microaerophiles or strict anaerobes, involved in sulfur metabolism (Mitchell et al., 2014). Some *Helicobacter* species are also associated with fecal pollution in coastal waters (Buccheri et al., 2019) and diverse gut microbiomes (Unno et al., 2018), including coastal invertebrates (Nakagawa et al., 2017). To the best of our knowledge, no *Helicobacter* species were reported as part of the salmon microbiome (Gerzova et al., 2014; Gajardo et al., 2016; Dehler et al., 2017; Fogarty et al., 2019; Gupta et al., 2019).

Common salmon gut microbiome bacterial families, which were also detected in significantly higher numbers at high impact sites but decreased toward moderate and low impact sites, were Lachnospiraceae (Fogarty et al., 2019), Ruminococcaceae (Gerzova et al., 2014; Dehler et al., 2017; Fogarty et al., 2019; Gupta et al., 2019), and Spirochaetaceae (Gupta et al., 2019). Also, representatives of the Flavobacteriaceae, which we identified with a significantly higher sequence read abundance in high impact sediments, are dominant members of the salmon gut microbiome (Fogarty et al., 2019; Gupta et al., 2019). However, due to their high metabolic versatility, this taxon group is generally widely distributed in marine sediments (Bowman, 2005; Bernardet and Nakagawa, 2006) and often associated with high organic enrichment (Bissett et al., 2008).

The relative abundance of the Caldilineaceae also decreased significantly with decreasing impact. Ape et al. reported a positive correlation of this family with organic enrichment and a negative correlation with oxygen concentration (Ape et al., 2019). During a remediation process of highly impacted aquafarm sediments, these authors showed a significant decrease of Caldilineaceae over time (Ape et al., 2019). Caldilineaceae are also important components of marine iron-oxidizing bacterial networks (Duchinski et al., 2019). The anaerobic degradation of high loads of organic matter in coastal sediments is coupled to iron metabolism as part of a “standard” redox cascade of terminal electron sinks (Canfield et al., 1993; Hyun et al., 2009). In addition to zinc, also iron is an additive in salmon feed, which may accumulate in sediments under salmon cages.

Members of the family Psychromonadaceae are typical for environments with highly abundant organic matter (Miyazaki and Nogi, 2014). In fish farm sediments, *Psychromonas* spp. were associated with a high degree of perturbation and an anaerobic carbohydrate metabolism (Miyazaki and Nogi, 2014; Keeley et al., 2020). Surprisingly, this family hardly finds attention in previous studies investigating bacterial community composition in impacted coastal sediments.

SRBs play a significant role in the biogeochemical cycling of sulfur and in the mineralization of organic matter. Therefore, it is not surprising that we found several SRB families as typical bacteria for cage site sediments, which agrees with several previous reports (Asami et al., 2005; Bissett et al., 2006; Kondo et al., 2008, 2012; Kawahara et al., 2009; Fodelianakis et al., 2014; Dowle et al., 2015; Stoeck et al., 2018a). Also, in sediments that are unrelated to aquaculture activities, SRBs were mentioned frequently as bioindicators of nutrient pollution, such as in eutrophic estuaries (Sun et al., 2013), polluted harbor sediments (Zhang et al., 2008), or marine costal sediments perturbed by a multitude of anthropogenic contaminations (Aylagas et al., 2017). Desulfobulbaceae are capable of sulfur disproportion, converting sulfur of intermediate oxidation to available sulfide (Finster et al., 1998; Frederiksen and Finster, 2004). Desulfarculaceae include only three genera, which utilize thiosulfate (all genera), sulfate (*Desulfarculus* and *Desulfocarbo*), and sulfite (*Desulfarculus*) as terminal electron acceptors and to be reduced to H_2_S (Galushko and Kuever, 2015). The latter contributes to the characteristic smell of most sediments from below salmon cages. Considering the metabolism of SRBs, carbon, hydrogen, and sulfur species in the deposition field of fish farms are ideal habitats for these bacteria. Desulfobacteraceae are able to metabolize a high variety of carbon sources and hydrogen (Isaksen and Teske, 1996; Schwartz and Friedrich, 2006) and are involved in a variety of processes regarding biodegradation of pollutants, organic matter turnover, and sulfur and carbon cycles (Zhang et al., 2008). They were also associated with metal-contaminated coastal sediments (Lanzen et al., 2020). Metals in aquaculture sediments may originate from dietary additives in feed (enriched with zinc and iron; Maage et al., 2001) and anti-fouling coatings (including copper) to prevent biofouling of cage structures, although this practice in Canada is giving way to automated net washing systems that use high pressure and saltwater. Clean cage structures are critical to maintain good water flow, ensuring high dissolved oxygen concentrations and maintaining fish health (Burridge et al., 2010; Hornick and Buschmann, 2017). Therefore, it is not surprising that high concentrations of (heavy) metals may occur in sediments under cages at fish farms treated with anti-fouling paints (Simpson et al., 2013; Nikolaou et al., 2014). Two further sulfur metabolizing taxon groups characteristic of high impact sites were Thiotrichaceae and Chromatiaceae. Some Thiotrichaceae species are forming bacterial mats that indicate organic pollution (Chet et al., 1975; Fenchel and Bernard, 1995; Elliott et al., 2006) and were assigned as an indicator for bad ecological status by Aylagas et al. (2017). Likewise, Chromatiaceae were reported as typical bacteria at aquaculture-impacted sites (Fodelianakis et al., 2014). Opitutaceae include species that are widely distributed in a variety of aquatic and terrestrial environments; and their ecological contributions remain to be understood (Rodrigues and Isanapong, 2014). A recent environmental sequencing study identified 16S rRNA gene sequences of Opitutaceae as members of “*Type 1* bioturbation lineages” associated with burrowing activities of marine benthic macroinvertebrates (Deng et al., 2020).

### Bacterial Families Typical of Moderate Impact Sites

**FIGURE 5:**
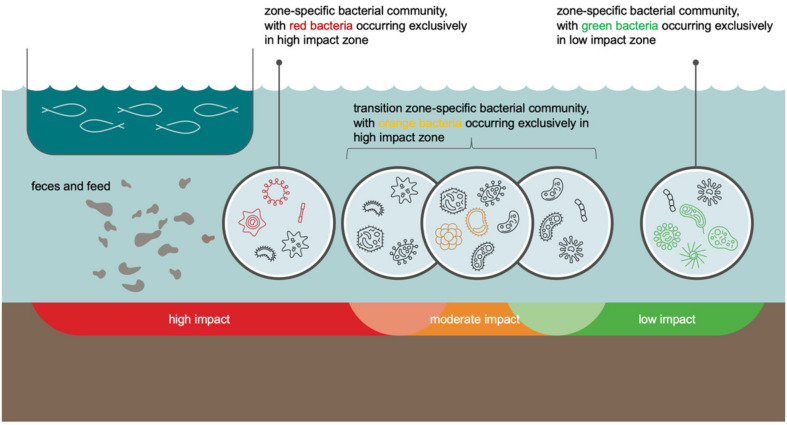
Conceptual graphic of changes in bacterial community composition along an aquaculture-related impact gradient (the “benthic cage aquaculture footprint”). The graphic represents especially the difficulties in identification of bacterial taxon groups that are typical of moderate impact conditions (orange benthic zone). This zone is a transition from high (in red) to low (in green) benthic impact and, thus, a melting pot that includes bacteria from all impact categories. Only few bacteria (marked in orange) have such a narrow ecological niche that they do not extend out of the transition zone into regions with high or low impact. On the other hand, some bacteria that are more typical of low or high impact conditions have an ecological tolerance to environmental conditions, which allows them to extend from regions of low or high impact, respectively, to regions of moderate impact. In environmental monitoring, relative abundance distributions of all bacterial taxon groups at each sampling site are considered, which, in concert, help to identify moderate impact conditions. Bacteria that are exclusive for high, moderate, and low impact conditions are visualized by their color code for each of these impact categories (red, orange, and green, respectively). Bacteria marked in black are bacteria that occur across two impact categories in the transition zones.

It was surprising that despite the highest average ASV richness and Shannon diversity being obtained for moderate impact sites, the number of bacterial discriminator families that are typical of this impact category was by far lower than that of high and low impact sites. As a possible explanation, we developed the following model, graphically shown in Figure 5: moderately impacted sites can be considered as a transition zone between two habitat types with notably distinct environmental conditions (high and low impact sites). Consequently, also bacterial communities do not change abruptly from the high or low impact zone to the moderate impact zone. Instead, the transition zone is a melting pot for bacterial families whose occurrence extends from the low and high impact zones into the moderate impact zone. This scenario explains the high bacterial family richness and Shannon index in this transition zone, as well as the low number of bacterial families that are discriminatory for the moderate impact zone. We assume that the few bacterial families that we identified as a specific indicator for the moderate impact zone have such narrow autecological properties that they are unable to establish in a noteworthy manner beyond the environmental conditions of the moderate impact zone.

Among the bacterial indicator families that were characteristic for sites with moderate aquaculture impact, the Vibrionaceae stood out due to their remarkably high relative abundances. Vibrionaceae are metabolically versatile bacteria degrading diverse hydrocarbon resources (Hedlund and Staley, 2001). Because of their metabolic versatility, it is not surprising that others have classified Vibrionaceae as indicators for bad ecological status in coastal sediments (Aylagas et al., 2017). Vibrionaceae commonly occur in benthic marine environments [reviewed in Vezzulli et al. (2009)]. Therefore, assigning an indicator function to this family may lead to erroneous environmental impact assessment. This is evidenced by a more detailed analysis of this family (Supplementary File 9), which demonstrates that lower taxon ranks within this family are better differential indicators for benthic disturbance. Vibrionaceae ASVs could be assigned to the to five species (*Aliivibrio fischeri*, *Photobacterium angustum*, *Photobacterium rosenbergii*, *Vibrio ichthyoenteri*, and *Vibrio rumoiensis*). *A. fischeri* and *V. ichthyoenteri* showed a significant preference for impacted sites. To the best of our knowledge, *A. fischeri* was thus far not associated with salmon (aquaculture). However, *Vibrio* spp., which have 96% sequence similarity to the 16S rRNA gene of *A. fischeri*, were isolated from sea-farmed Atlantic salmon with skin ulcer (Lunder et al., 2000). *V. ichthyoenteri* was isolated from the hindgut of farmed Atlantic salmon (Hatje et al., 2014), explaining its higher abundances in sediments impacted by salmon farming. *V. rumoiensis* and *P. rosenbergii* dominated at moderately impacted sites. The former is a non-pathogenic free-living facultative psychrophilic *Vibrio* species with a high catalase activity (Yumoto et al., 1999), while the latter is generally associated with undefined diseases in sponges (Moi et al., 2017). However, we note that due to the tremendous diversity and versatile lifestyles of Vibrionaceae species and the difficulties of accurate species assignments to ASVs, further detailed investigations are necessary to move beyond these findings and to solidly interpret our results. Until then, ASVs are most likely our best option at hand if we are to assign Vibrionaceae to specific environmental impact categories.

Acidaminobacteraceae, another family characteristic of the moderate impact zone, were previously associated with marine finfish aquaculture. For example, they were reported as a major contributor to the differences in benthic bacterial communities between mariculture and non-mariculture sites (Li et al., 2013; Ape et al., 2019). This bacterial family is important for the anaerobic degradation of organic matter in marine systems (Schwarz et al., 2008). Species of the family Shewanellaceae, which are widely distributed in marine habitats (Dikow, 2011), are facultative anaerobes, which under oxygen-depletion use, for example, thiosulfate, sulfite, or sulfur as an electron acceptor (Burns and DiChristina, 2009). Moreover, *Shewanella* species are well-known as efficient players in the effective removal of various complex organic and inorganic pollutants (bioremediation) (Zou et al., 2019).

### Bacterial Families Typical of Low Impact Sites

The notably different environmental conditions at low impact far-field sites are indicated through specific bacterial families, which differ notably in their metabolic properties as compared with the sites with high impact resulting from aquaculture. As a rule, parameters that are notably different between aquaculture impacted sites and non-impacted sites include the sediment sulfide and oxygen concentrations, pH values, and nutrients (Janowicz and Ross, 2001; Wildish et al., 2001; Brooks and Mahnken, 2003; Carroll et al., 2003; Hargrave et al., 2008), all of which govern the community composition of bacteria (McCaig et al., 1999; Asami et al., 2005; Bissett et al., 2006; Dowle et al., 2015). Rhodospirillaceae as characteristic family of low impact sites under study are inhibited by high sulfide concentrations (van Niel, 1944; Pfennig, 1967). Pirellulaceae react sensitively to lower pH values, as are typical of near-field sites (Usharani, 2019), and Planctomycetaceae dislike eutrophic conditions that can be found in closer vicinity to salmon cages (Li et al., 2017). Accordingly, these bacterial taxon groups are common members of natural benthic bacterial communities (Lanzen et al., 2020). Also, the obligate aerobe Flammeovirgaceae, which are chemoheterotrophic microbes and very common in marine environments (Yoon et al., 2011), prefer the oxygenated, less organically enriched sediments of far-field sites. While aquaculture impacted sites were typically associated with sulfur-metabolizing bacteria, nitrogen-metabolizing bacteria were more characteristic of low impact sites. Nitrosomonadaceae are ammonia-oxidizing bacteria that require oxygen (Prosser et al., 2014). In agreement with our study, Moncada et al. (2019) found a significantly higher abundance of Nitrosomonadaceae in off-cage sediments compared with fish cage sediments of Philippine mariculture. Nitrospinaceae are obligate marine aerobic chemolithoautotrophic nitrite-oxidizing bacteria (Lücker and Daims, 2014), which explains their avoidance of organically enriched sediments. Not surprisingly, *Nitrospina*-like organisms are frequently detected by molecular methods as regular members of bacterial communities in oxic but occasionally also in suboxic marine environments (Lücker and Daims, 2014). Nitrospiraceae contain chemolithotrophic aerobic nitride-oxidizing bacteria (Daims, 2014). In a previous study, which developed a multitrophic DNA-based metabarcoding tool for benthic monitoring of aquaculture farms, Pochon et al. (2017) identified *Nitrospira* species as characteristic bacteria of undisturbed, organically unenriched conditions. In the same study, as well as in a study by Keeley et al. (2018), also Thermodesulfovibrionaceae, which can utilize nitrate, sulfite, thiosulfate, and sulfate as terminal electron acceptors, were characterized as indicative of far-field, unenriched sites. Thermodesulfovibrionaceae and Nitrospiraceae often co-occur in benthic marine nitrogen-metabolizing communities (Baskaran et al., 2020). Likewise, Ectothiorhodospiraceae include widely distributed aerobic and anaerobic bacteria capable of nitrite and sulfur oxidation (Oren, 2014), which were previously reported as indicators of high-to-good environmental quality in coastal sediments (Aylagas et al., 2017). Accordingly, Stoeck et al. (2018a) as well as Keeley et al. (2018) reported Ectothiorhodospiraceae as typical members of undisturbed far-field sites in coastal aquaculture areas. Hyphomicrobiaceae are typical natural sediment bacterial communities (Mills et al., 2008). Not surprisingly, they were previously identified as sensitive to environmental perturbances and, thus, indicate a good ecological status (Aylagas et al., 2017). In agreement with our results, Alteromonadaceae were previously identified as indicators of good ecological status in coastal marine sediments, due to their sensitivity to environmental stressors (Dowle et al., 2015; Aylagas et al., 2017). Members of this obligate aerobe, heterotroph, metabolically diverse, and, in marine environments, widely distributed family, however, were also associated with nutrient-rich conditions and hydrocarbon degradation (López-Pérez and Rodriguez-Valera, 2014). In summary of these results, we can conclude that the sensitivity to impact conditions is probably the strongest selection criterion for the bacterial taxa characteristic of benthic reference conditions.

Several families that were characteristic for reference site conditions in the samples under study have contrasting indicator qualities in the literature. The family Piscirickettsiaceae is widely distributed in undisturbed sediments (Gribben et al., 2017) but also associated with organic pollution (Zhang et al., 2016; Wang et al., 2018). The family contains phylogenetically related genera with highly diverse characteristics, making them very different from one another. The genus *Piscirickettsia* comprises a single species called *Piscirickettsia salmonis*, a Gram-negative facultative intracellular fish pathogen that significantly affects the salmon industry. Since its first isolation in Chile in 1989, the bacterium has been reported in Norway, Scotland, Greece, Canada, and the United States, among others (Marshall et al., 2014). While Desulfuromonadaceae (iron-reducing bacteria and SRBs) were more abundant in unperturbed sediments in studies of Dowle et al. (2015) and Aylagas et al. (2017), the same family was indicating perturbed coastal sediment conditions (Lanzen et al., 2020), pollution from oil drilling in offshore sediments (Nguyen et al., 2018), and organically enriched sediments (Julies Elsabé et al., 2012; Aranda et al., 2015). Likewise, Syntrophobacteraceae were found in undisturbed sediments as well as at sites with high organic enrichment (Choi et al., 2018). Marinicellaceae are associated with anthropogenically disturbed marine coastal ecosystems (Barbato et al., 2016; Won et al., 2017), while others reported a sensitive reaction (significant decrease in abundance) after exposing undisturbed pristine sediment to hydrocarbon pollution (Koo et al., 2015). Also, Fusobacteriaceae, even though occurring in higher relative abundances at low impact sites compared with moderate and high impact sites, are most likely not a typical low impact taxon group. This family includes free-living marine benthic species as well as species living in the intestines of diverse marine invertebrates and fish, including, but not restricted to, salmon (Olsen, 2014; Dehler et al., 2017; Zoqratt et al., 2018; Yukgehnaish et al., 2020). Accordingly, it is not surprising that others have reported Fusobacterium as a characteristic member of the benthic bacterial community in sediments highly impacted by salmon aquaculture, while missing from moderately disturbed sampling sites (Verhoeven et al., 2018). A similar observation was reported for a Scottish salmon farm, but only from one of two sample replicates (Dully et al., 2020). These results further support the argument that in some cases, the family rank is insufficient to assign clear indicator properties to bacterial taxa, and higher taxonomic resolution for ASVs is required (Cordier, 2020). This, however, makes amending of bacterial reference sequence databases on lower taxonomic levels mandatory (Beng and Corlett, 2020), if we are to better exploit metabarcoding data of natural bacterial communities in ecology and biomonitoring.

## Conclusion

We have identified benthic bacterial taxon groups that are indicative for the degree of aquaculture-related impact on coastal ecosystems across large geographic scales. This is best explained by the combination of two basic principles in microbiological ecology: i) most bacteria have high global dispersal capabilities (Finlay, 2002; Martiny et al., 2006; Yamaguchi et al., 2012). ii) The evolution of bacterial communities is ecology-driven (O’Malley, 2008), implying that only specifically adapted organisms thrive and proliferate in a particular environment (Fuhrman, 2009). These two basic principles were summarized already in 1934 in the famous microbiological tenet “everything is everywhere, but, the environment selects” by Baas-Becking (1934). The main environmental drivers for the bacterial indicators identified in this study are likely to be predominantly typical aquaculture-related impacts (organic enrichment, metals), while indicators of unimpacted benthic sediments are mostly selected by their sensitivity toward these stressors and acclimation to natural levels. Our findings may provide a solid basis, which could be further developed into bioindicators for compliance monitoring and for aquafarm management. Bacterial taxon groups identified in our study, which are indicative for specific degrees of aquaculture impact on the benthic ecosystems, can now be chosen as a target for the development of specific PCR primers for a fast, sequencing-independent, screening of environmental samples in quantitative PCR assays. Such technologies could then be further developed for routine application in the field.

## Data Availability Statement

The datasets presented in this study can be found in online repositories. The names of the repository/repositories and accession number(s) can be found in the article/Supplementary Material.

## Author Contributions

LF: investigation, formal analysis, data curation, visualization, and writing first draft. VD: investigation, formal analysis, and visualization. DF: formal analysis and validation. NK and SR: resources, validation, data interpretation, and writing. OL: methodology, validation, data interpretation, and writing. XP: resources, validation, data interpretation, writing, and funding acquisition. TW: validation, data interpretation, and writing. TS: conceptualization, methodology, validation, writing, supervision, project administration, and funding acquisition. All authors contributed to the article and approved the submitted version.

## Conflict of Interest

The authors declare that the research was conducted in the absence of any commercial or financial relationships that could be construed as a potential conflict of interest.

## Supplementary Material

Supplementary File 1 Information on sampling sites.

Supplementary File 2 Rarefaction curves of all 138 samples under study.

Supplementary File 3 Overview of evolution of sequence data from original fastq files to final data used in downstream analyses for each individual salmon farm under study.

Supplementary File 4 Mean decrease accuracy and corresponding Gini coefficient for each of the 88 bacterial families identified to predict the degree of aquaculture-related impact using Random Forest.

Supplementary File 5 Bacterial families that are unique for either high (*n* = 6), moderate (*n* = 5) or low (*n* = 23) aquaculture-related impact sites.

Supplementary File 6 Bacterial families that were ubiquitously distributed across all three aquaculture-related impact categories (high, moderate, and low impact).

Supplementary File 7 Distribution of detected bacterial families across the different biogeographic regions samples in this study. The last column (perm_test_signif) shows the impact category, in which an individual family was detected with significantly higher occurrence. This is only shown for the 32 bacterial families that were subjected to significance testing (see “Materials and Methods” section).

Supplementary File 8 Comparison of RF predictions (left) and variable importance (right) after fourth root and centered log-ratio transformation of datasets. In both cases, the family rank achieved the best RF predictions and also the most important bacterial families are highly congruent in a comparison of both approaches.

Supplementary File 9 Taxonomic assignment of Vibrionaceae-ASVs to species rank. A high proportion could not be assigned to species rank (category “unassigned”). Relative abundance numbers refer to the complete sequence data set analyzed in this study (see Supplementary File 3).
