# Supplementary figures and images for: Global Trends of Benthic Bacterial Diversity and Community Composition Along Organic Enrichment Gradients of Salmon Farms
Source: Front Microbiol. 2021 Apr 29;12:637811. doi: 10.3389/fmicb.2021.637811 (PMC8116884; doi:10.3389/fmicb.2021.637811)

## Slide 1
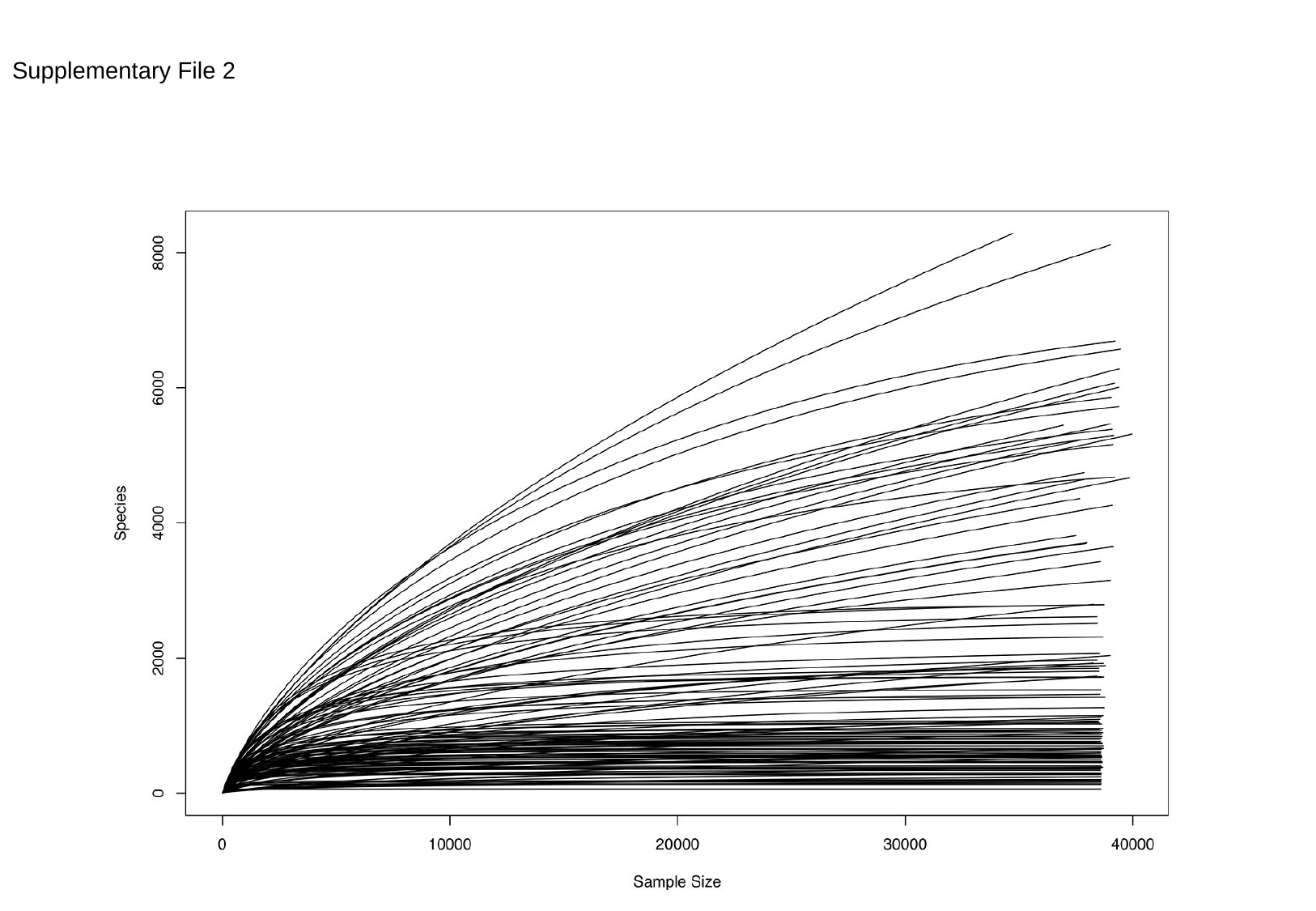

# Supplementary File 2

Supplement: Supplementary File 2 — Rarefaction curves of all 138 samples under study. [file Presentation_1.PPTX]

## Slide 1
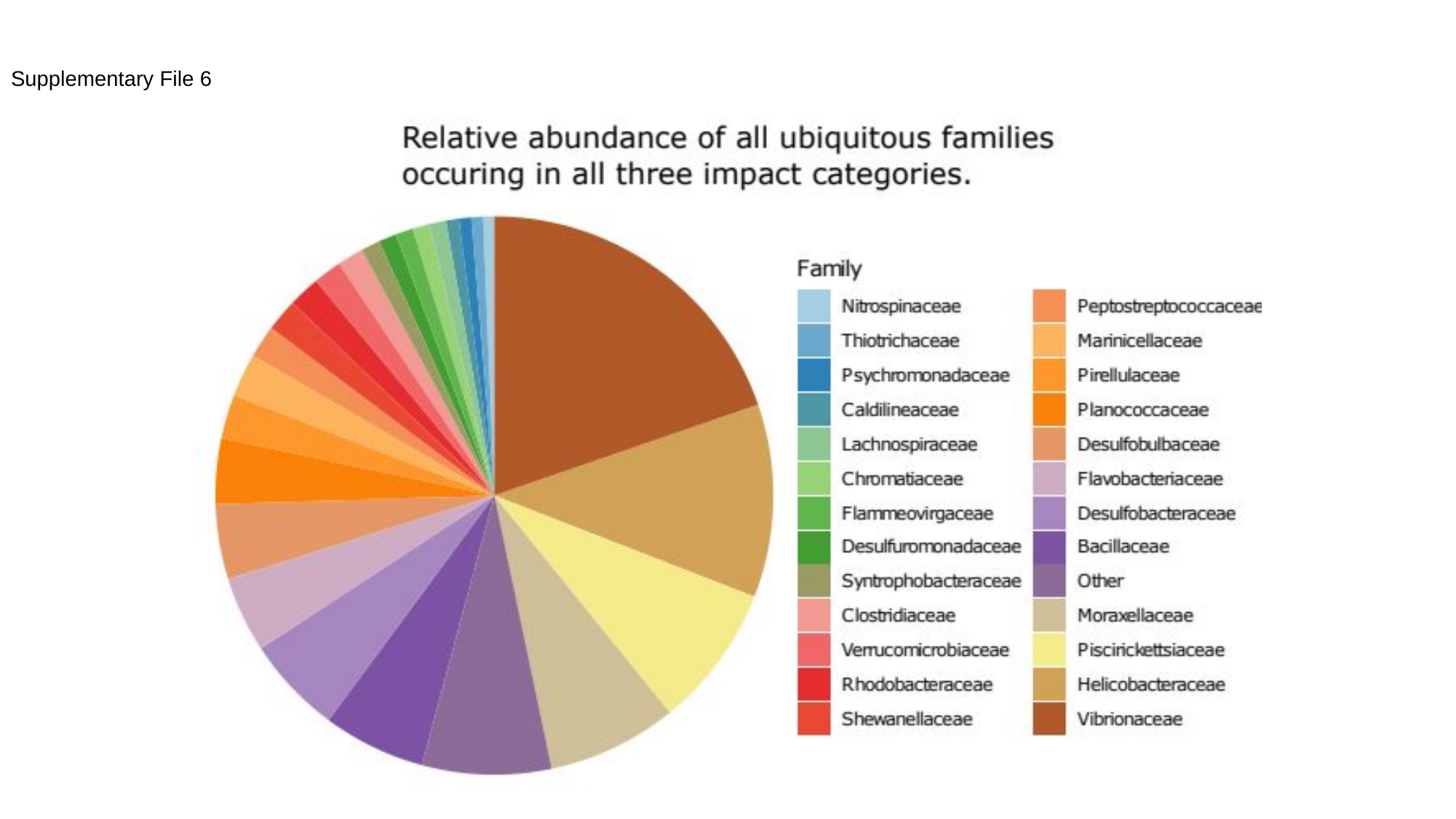

# Supplementary File 6

Supplement: Supplementary File 6 — Bacterial families that were ubiquitously distributed across all three aquaculture-related impact categories (high, moderate, and low impact). [file Presentation_2.PPTX]

## Slide 1
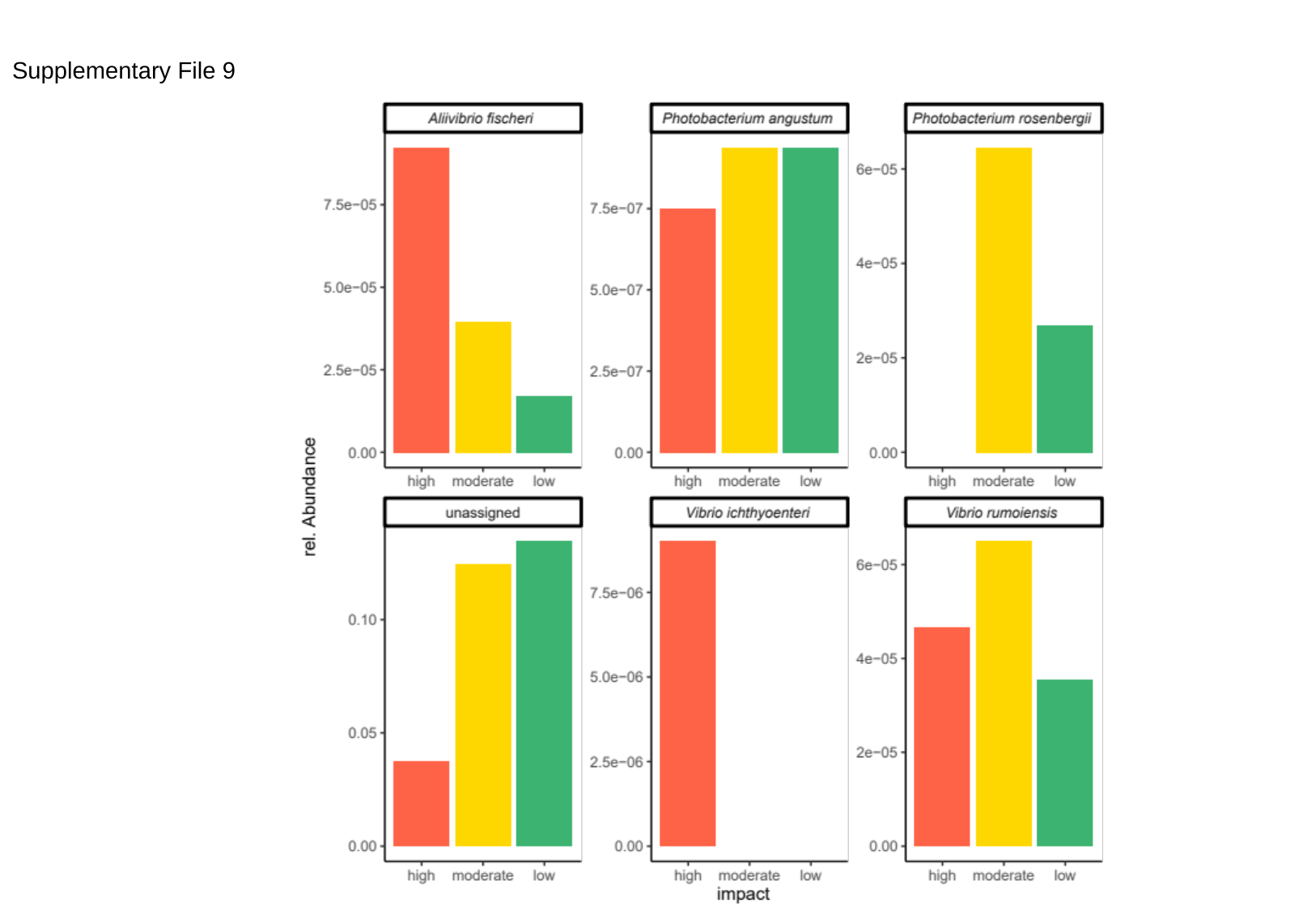

# Supplementary File 9

Supplement: Supplementary File 9 — Taxonomic assignment of Vibrionaceae-ASVs to species rank. A high proportion could not be assigned to species rank (category “unassigned”). Relative abundance numbers refer to the complete sequence data set analyzed in this study (see Supplementary File 3). [file Presentation_4.PPTX]
