# Supplementary material for: Global Trends of Benthic Bacterial Diversity and Community Composition Along Organic Enrichment Gradients of Salmon Farms
Source: Front Microbiol. 2021 Apr 29;12:637811. doi: 10.3389/fmicb.2021.637811 (PMC8116884; doi:10.3389/fmicb.2021.637811)
Supplement: Supplementary File 8 — Comparison of RF predictions (left) and variable importance (right) after fourth root and centered log-ratio transformation of datasets. In both cases, the family rank achieved the best RF predictions and also the most important bacterial families are highly congruent in a comparison of both approaches. [file Presentation_3.PPTX]

## Slide 1
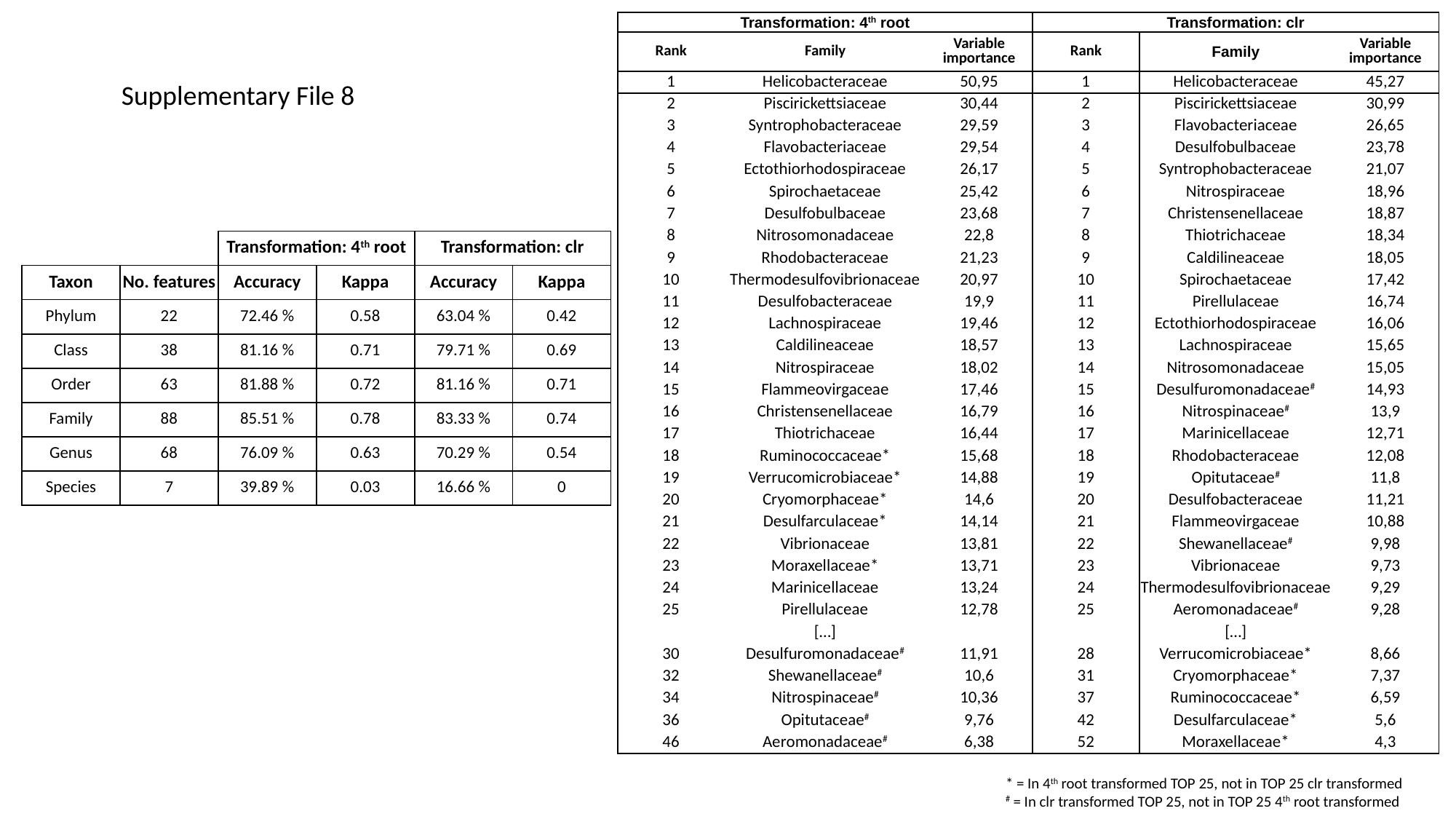

| Transformation: 4th root | Transformation\_ 4th root | | Transformation: clr | Transformation: clr | |
| --- | --- | --- | --- | --- | --- |
| Rank | Family | Variable importance | Rank | Family | Variable importance |
| 1 | Helicobacteraceae | 50,95 | 1 | Helicobacteraceae | 45,27 |
| 2 | Piscirickettsiaceae | 30,44 | 2 | Piscirickettsiaceae | 30,99 |
| 3 | Syntrophobacteraceae | 29,59 | 3 | Flavobacteriaceae | 26,65 |
| 4 | Flavobacteriaceae | 29,54 | 4 | Desulfobulbaceae | 23,78 |
| 5 | Ectothiorhodospiraceae | 26,17 | 5 | Syntrophobacteraceae | 21,07 |
| 6 | Spirochaetaceae | 25,42 | 6 | Nitrospiraceae | 18,96 |
| 7 | Desulfobulbaceae | 23,68 | 7 | Christensenellaceae | 18,87 |
| 8 | Nitrosomonadaceae | 22,8 | 8 | Thiotrichaceae | 18,34 |
| 9 | Rhodobacteraceae | 21,23 | 9 | Caldilineaceae | 18,05 |
| 10 | Thermodesulfovibrionaceae | 20,97 | 10 | Spirochaetaceae | 17,42 |
| 11 | Desulfobacteraceae | 19,9 | 11 | Pirellulaceae | 16,74 |
| 12 | Lachnospiraceae | 19,46 | 12 | Ectothiorhodospiraceae | 16,06 |
| 13 | Caldilineaceae | 18,57 | 13 | Lachnospiraceae | 15,65 |
| 14 | Nitrospiraceae | 18,02 | 14 | Nitrosomonadaceae | 15,05 |
| 15 | Flammeovirgaceae | 17,46 | 15 | Desulfuromonadaceae# | 14,93 |
| 16 | Christensenellaceae | 16,79 | 16 | Nitrospinaceae# | 13,9 |
| 17 | Thiotrichaceae | 16,44 | 17 | Marinicellaceae | 12,71 |
| 18 | Ruminococcaceae\* | 15,68 | 18 | Rhodobacteraceae | 12,08 |
| 19 | Verrucomicrobiaceae\* | 14,88 | 19 | Opitutaceae# | 11,8 |
| 20 | Cryomorphaceae\* | 14,6 | 20 | Desulfobacteraceae | 11,21 |
| 21 | Desulfarculaceae\* | 14,14 | 21 | Flammeovirgaceae | 10,88 |
| 22 | Vibrionaceae | 13,81 | 22 | Shewanellaceae# | 9,98 |
| 23 | Moraxellaceae\* | 13,71 | 23 | Vibrionaceae | 9,73 |
| 24 | Marinicellaceae | 13,24 | 24 | Thermodesulfovibrionaceae | 9,29 |
| 25 | Pirellulaceae | 12,78 | 25 | Aeromonadaceae# | 9,28 |
| | […] | | | […] | |
| 30 | Desulfuromonadaceae# | 11,91 | 28 | Verrucomicrobiaceae\* | 8,66 |
| 32 | Shewanellaceae# | 10,6 | 31 | Cryomorphaceae\* | 7,37 |
| 34 | Nitrospinaceae# | 10,36 | 37 | Ruminococcaceae\* | 6,59 |
| 36 | Opitutaceae# | 9,76 | 42 | Desulfarculaceae\* | 5,6 |
| 46 | Aeromonadaceae# | 6,38 | 52 | Moraxellaceae\* | 4,3 |
Supplementary File 8
| | | Transformation: 4th root | | Transformation: clr | |
| --- | --- | --- | --- | --- | --- |
| Taxon | No. features | Accuracy | Kappa | Accuracy | Kappa |
| Phylum | 22 | 72.46 % | 0.58 | 63.04 % | 0.42 |
| Class | 38 | 81.16 % | 0.71 | 79.71 % | 0.69 |
| Order | 63 | 81.88 % | 0.72 | 81.16 % | 0.71 |
| Family | 88 | 85.51 % | 0.78 | 83.33 % | 0.74 |
| Genus | 68 | 76.09 % | 0.63 | 70.29 % | 0.54 |
| Species | 7 | 39.89 % | 0.03 | 16.66 % | 0 |
* = In 4th root transformed TOP 25, not in TOP 25 clr transformed
# = In clr transformed TOP 25, not in TOP 25 4th root transformed
